# Nearby Construction Impedes the Progression to Overt Autoimmune Diabetes in NOD Mice

**DOI:** 10.1155/2013/620313

**Published:** 2013-04-18

**Authors:** Erin E. Hillhouse, Roxanne Collin, Geneviève Chabot-Roy, Marie-Josée Guyon, Nathalie Tessier, Maryse Boulay, Patricia Liscourt, Sylvie Lesage

**Affiliations:** ^1^Maisonneuve-Rosemont Hospital Research Center, Montréal, QC, Canada H1T 2M4; ^2^Department of Microbiology and Immunology, University of Montreal, Montréal, QC, Canada H3C 3J7; ^3^Centre de Recherche du Centre Hospitalier de l'Université de Montréal, Montréal, QC, Canada H2L 2W5

## Abstract

Construction nearby animal houses has sporadically been reported to affect various aspects of animal health. Most of the reports have focussed on the impact on stress hormone levels and the hypersensitivity of animals relative to humans. There has also been an anecdotal report on the impact of construction on autoimmune diabetes in NOD mice. Here, we describe that nearby construction significantly impedes the progression to overt diabetes in female NOD mice offspring. We demonstrate that this was not due to a genetic drift or to particularities associated with our specific mouse colony. Interestingly, although the glycemia levels remained low in mice born from mothers subject to construction stress during gestation, we detected an active autoimmune reaction towards pancreatic islet cells, as measured by both the degree of insulitis and the presence of insulin autoantibody levels in the serum. These results suggest that the external stress imposed during embryonic development does not prevent but significantly delays the autoimmune process. Together, our findings emphasize the impact of surrounding factors during *in vivo* studies and are in agreement with the hypothesis that both environmental and genetic cues contribute to autoimmune diabetes development.

## 1. Introduction

The NOD mouse was developed in the 1970s in Japan and has since become the animal model of choice to investigate the genetic, cellular, and molecular mechanisms involved in the development of autoimmune diabetes [1]. This inbred strain spontaneously develops an autoimmune reaction towards pancreatic islet antigens, destroying the insulin-producing pancreatic beta cells. The spontaneous disease process in the NOD mouse parallels that observed in humans and, consequently, the NOD mouse model provides an important tool to dissect and better understand the complex pathophysiological process leading to disease onset [2, 3]. 

Two main factors are known to contribute to disease susceptibility of this complex trait, namely, genetic and environmental factors. First, autoimmune diabetes is a complex genetic trait, with over 50 genetic loci contributing to disease susceptibility [4, 5]. Interestingly, a direct parallel can be made between most of the diabetes susceptibility genetic risk factors currently identified in humans and mice [6]. However, a notable difference is that although the prevalence of autoimmune diabetes in humans is not influenced by gender, the incidence in female NOD mice is consistently higher than that of male mice [1]. Nevertheless, the NOD strain constitutes a valid model to investigate the pathophysiology and the genetic susceptibility towards the spontaneous occurrence of autoimmune diabetes. Second, the contribution of environmental factors to diabetes susceptibility in humans is less well understood and include, but are not limited to, viral infections, diet and epigenetic modifications [7, 8]. 

Interestingly, environmental factors are not only at play in defining susceptibility to autoimmune diabetes in humans but also exhibit a major impact on susceptibility to disease in the inbred NOD strain. Indeed, the incidence of diabetes is known to vary depending on the barrier status of the animal house, where the incidence of disease is the highest in specific pathogen-free facilities relative to conventional facilities [9–11]. The type of diet provided to NOD mice or the ambient temperature in which the mouse colonies are kept can both impact the incidence of disease [12–15]. Finally, the Jackson Laboratory has reported modest changes in the incidence of disease in their large NOD mouse cohorts due to alterations in light cycles or to nearby construction and earthquake tremors (http://type1diabetes.jax.org/images/fine-mapping/1976%20cumulative%20inc.jpg).

We currently hold a NOD mouse colony in our specific pathogen-free facility, for which we routinely monitor the incidence of diabetes in female mice. These data serve as a control for ongoing experiments, wherein we administer different products to female NOD mice to determine the impact of these products on the pathophysiology of the disease as well as the incidence of diabetes [16]. In our small animal cohort, we show that nearby construction significantly delayed diabetes onset in NOD mice and almost completely impeded the progression to overt diabetes. We also demonstrate that this is not a consequence of a genetic drift in our NOD mouse colony. The results emphasize the importance of considering the surrounding environment when performing experiments in animal models.

## 2. Methods

### 2.1. Ethics Statement

All experiments were performed in line with the rules and regulations of the Canadian Council for Animal Protection, and the experimental procedure was approved by the Maisonneuve-Rosemont Hospital Animal Care Committee.

### 2.2. Mice

NOD mice from the NOD/LtJ colony were purchased from Jackson Labs in 2006 and were maintained by intercrossing nondiabetic 6-week-old male and female mice at the Hôpital Maisonneuve-Rosemont (HMR) specific pathogen-free facility. Routine microbiological monitoring of dirty bedding exposed sentinel animals was performed for the following murine pathogens: mouse hepatitis virus, Sendai virus, pneumonia virus of mice, reovirus-3, Theiler's murine encephalomyelitis virus, *Mycoplasma pulmonis*, mouse parvovirus, mice minute virus, mouse rotavirus, murine norovirus, *Helicobacter genus, Helicobacter bilis, Helicobacter hepaticus*, *Bordetella bronchiseptica, Corynebacterium kutscheri, Klebsiella oxytoca, Klebsiella pneumoniae, Pasteurella multocida, Pasteurella pneumotropica (Heyl and Jawetz), Pseudomonas aeruginosa, Staphylococcus aureus, Streptococcus pneumonia, Beta Strep* spp., *Beta Strep*. sp.*-Group B, Beta Strep*. sp.*-Group G, Salmonella, Citrobacter rodentium, Clostridium piliforme. *The colony was also free of the following endo- and ectoparasites: lice, mites, *Aspicularis tetraptera*, *Syphacia murisSyphacia obvelata*, *Chilomastix* sp. *Entamoeba* sp., *Giardia, Hexamastix* sp., *Monocercomonoides* sp., *Retortamonas* sp., *Spironucleus* sp., and *Trichomonads*. The colony remained free of these specific pathogens for the period of interest, aside from one report of *Entamoeba* sp. in July 2011. Animals were kept under a photoperiod of 14 hours of light/10 hours of darkness and ambient temperatures set at a range of 21°C to 24°C. Animals had unlimited access to distilled and acidified water and standard rodent diet (2018 Teklad Global 18% Protein Rodent Diet, or 2019 Teklad Global 19% Protein Extruded Rodent Diet, Harlan Laboratories Inc., Indianapolis, IN, USA). Mice were maintained in polycarbonate individually ventilated cages with hardwood bedding (7090A Teklad Aspen SaniChips, Harlan Laboratories Indianapolis Inc., IN, USA).

The breeding couples have been renewed, on average, every 14 weeks in an attempt to minimize potential genetic drifts [17]. The incidence of diabetes was monitored when the colony was at the HMR F11 to F13 generations. This mouse colony is hereafter referred to as NOD/LtJ-HMR. NOD mice were also purchased from the NOD/ShiLtJ colony at Jackson Labs in January 2011 and were maintained at the HMR under the same conditions as the NOD/LtJ-HMR colony. All our NOD mouse colonies were maintained by on-site breeding, in the same barrier status from 2006 to 2012, with no change in the housing conditions, husbandry practices, feed, or water. No embryo transfer or cross-fostering was undertaken in these colonies.

### 2.3. Construction

Asphalt and part of the sidewalk were repaired from September 15 to 18, 2010. The animal house where all the NOD mice are kept is located in the basement immediately adjacent to this construction site. At that time, we held three breeder pairs, for which the details are provided in Table 1.

### 2.4. Monitoring Diabetes Incidence

Diabetes incidence was monitored daily for overt signs of diabetes (wet cage, hunched posture) and every two weeks for urine glucose levels using Diastix (Bayer) starting at between 8 to 10 weeks of age. Mice were called diabetic upon two consecutive positive urine glucose readings. Blood glucose measurements >12 mM were used to confirm diabetes. Mice presenting with >12 mM of blood glucose were sacrificed within one week. All other mice were sacrificed at 32 weeks of age. At sacrifice, the pancreas was collected and frozen in Optimal Cutting Temperature Compound (OCT, Fisher) and the serum was collected and stored at −80°C. 

### 2.5. Insulitis

Frozen pancreases were cut to 7 *μ*m sections, pressed against slides, fixed in acetone, and stored overnight at 4°C prior to hematoxylin and eosin staining. Three to six nonconsecutive sections of pancreas were analysed per mouse. The number of islets as well as the degree of insulitis was quantified according to the following scale (see Figure 4): 0, no infiltration; 1, lymphocytes surrounding the islets; 2, lymphocytes surrounding the islets and break of barrier (less than 50% infiltration); 3, lymphocytes within the islets (over 50% infiltration); 4 extensive lymphocytic infiltrate with few or no detectable pancreatic islet cells. 

### 2.6. Insulin Autoantibodies (IAA)

The serum levels of IAA were measured by ELISA in a protocol adapted from previous work [18–20]. Insulin (Insulin B (9–23), Anaspec) was immobilized onto microwells in 96 well plates. As a negative control, half of each serum sample was previously incubated with insulin for 7 days. All serum samples (preincubated with insulin or not) were diluted 1/10 and were added to the insulin-coated microwells. Horseradish peroxidase-labeled polyclonal goat anti-mouse IgG (Biolegend) followed by TMB substrate solution (Biolegend) was used to quantify insulin-specific IgG antibodies. The optical density (OD) is directly proportional to the concentration of IAA in the sample. All samples were run in duplicate. The level of IAA in the sample = (average of sample OD) − (average of background control OD).

### 2.7. Statistics

Log rank Mantel-Cox tests were performed using GraphPad Prism 5 to determine the statistical significance of the difference in the incidence of diabetes.

## 3. Results and Discussion

**Figure 1:**
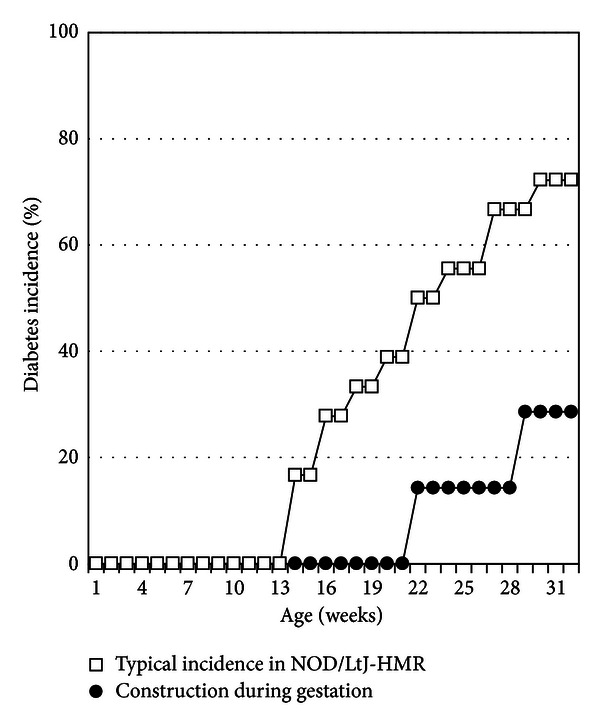
Nearby construction during the gestation period impacts diabetes onset and cumulative incidence in offspring. Depicted is a typical cumulative incidence of diabetes for eighteen female NOD/LtJ-HMR mice (open squares). Two NOD/LtJ-HMR female mice were in gestation while asphalt and sidewalk repairs were being carried out immediately outside the facility. The incidence of diabetes was closely monitored in the seven female offspring from these mothers (closed circles), where the onset is observed at 22 weeks of age and the cumulative incidence reaches approximately 30% at 32 weeks of age. Log-rank test, *P* value < 0.05.

Jackson Labs had previously reported that nearby construction partially affected the diabetes incidence in their NOD/LtJ colony in 2006 (http://type1diabetes.jax.org/images/fine-mapping/1976%20cumulative%20inc.jpg). Therefore, as we were aware of nearby construction upon undertaking a new diabetes incidence study in September 2010, we closely monitored the diabetes onset and incidence in the female NOD mouse experimental control group. Notably, at the HMR-specific pathogen-free animal house facility, diabetes onset in female NOD mice from the NOD/LtJ-HMR colony is typically between 12 to 14 weeks of age, and the incidence of diabetes reaches approximately 70%–80% at 32 weeks of age (Figure 1). 

Two of the three female NOD mice, that had been placed in breeding pairs, were in gestation during the construction period—September 15 to 18, 2010 (Table 1). We monitored the incidence of diabetes in the seven female offspring born in October 2010 from these mothers. We observed a delay in diabetes onset, which now initiated at 22 weeks of age for female NOD mice born from mothers subject to construction stress during gestation (Figure 1). Moreover, we found a significantly reduced cumulative incidence of diabetes, where only two of the seven female NOD mice developed diabetes within 32 weeks in the NOD/LtJ-HMR mouse colony (Figure 1).

Notably, the incidence of diabetes in Figure 1 was monitored in female NOD mice inbred for 11 to 13 generations in a relatively small animal house cohort, generally comprised of 3 to 5 breeder pairs. Although breeder replacement is performed on average every 14 weeks to prevent potential genetic selection for diabetes resistance, we could not entirely exclude the possibility of a genetic drift in the NOD/LtJ-HMR mouse colony [17, 21–23]. Indeed, as diabetes onset normally occurs between 12 to 14 weeks of age in female NOD mice of the NOD/LtJ-HMR mouse colony, and since we maintained a few breeder pairs beyond that time point, there is a risk that we selected mice carrying a genetic polymorphism conferring resistance to diabetes. 

**Figure 2:**
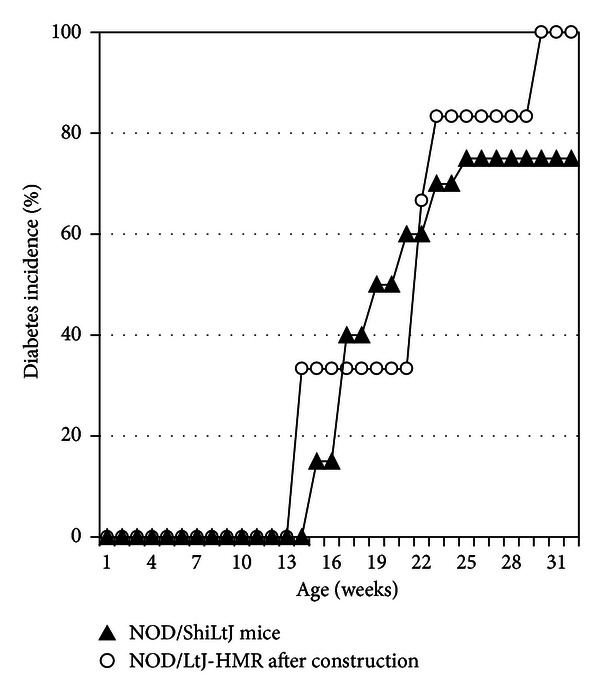
The lower incidence of diabetes is not due to a genetic drift in our colony. The onset of diabetes was monitored in six female NOD mice from the NOD/LtJ-HMR mouse colony born more than two months after the construction had been completed (open circles) as well as from twenty female NOD/ShiLtJ mice bought from Jackson Labs and maintained at the HMR facility (closed triangles). The cumulative diabetes incidence shows no difference between these two colonies. Log-rank test, *P* value = 0.4142.

We thus went on to test the hypothesis that the diabetes onset was delayed and that the incidence was reduced in the NOD/LtJ-HMR mouse colony as a consequence of a genetic drift. We undertook a second diabetes incidence study with six female NOD mice of the NOD/LtJ-HMR colony born in December 2010, more than two months after the construction had been completed. In addition, we purchased twenty 7-week-old female NOD mice from the Jackson Labs NOD/ShiLtJ mouse colony and maintained them in the same conditions as the mice from the NOD/LtJ-HMR colony. The onset of diabetes for female NOD mice from both the NOD/LtJ-HMR and NOD/ShiLtJ mouse colonies was between 12–15 weeks of age and the cumulative incidence of diabetes also reached 70–100% in both mouse colonies by 32 weeks of age (Figure 2). Together, our data demonstrate that the decrease in disease onset and cumulative incidence observed in the female NOD mice born in October 2010 was not due to a genetic drift in our mouse colony and is likely attributable to the effect of nearby construction. 

**Figure 3:**
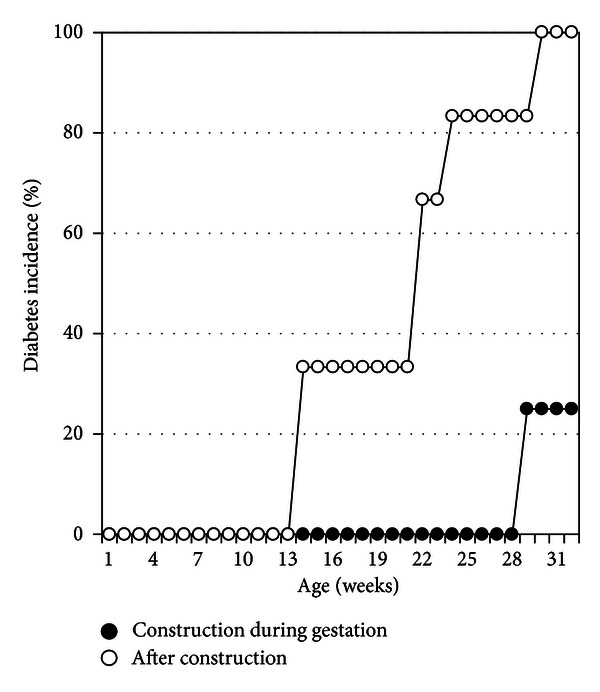
Nearby construction affects diabetes onset and cumulative incidence in NOD mice born from the same breeder pair. The cumulative incidence of diabetes is compared for female NOD mice born from the same breeder pair at different times, namely, those who nearby construction occurred during their embryonic development (closed circles, *n* = 4) and those born over two months after the construction event (open circles, *n* = 6). Log-rank test, *P* value < 0.05.

Of interest, all of the six female NOD mice of the NOD/LtJ-HMR colony from Figure 2 were born from one of the two original breeders, wherein the mothers had been subject to construction stress during the gestation two months before. We thus opted to directly compare the diabetes incidence from the four female NOD mice born in early October 2010 (i.e., subject to construction stress during their embryonic development) to the six female NOD mice born in December 2010, where all 10 offspring are of the same breeder pair. We again find a statistical difference in the incidence of diabetes as well as a delay in disease onset (Figure 3). These results demonstrate that the biological effects causing alterations in diabetes susceptibility due to construction stress are rapidly dissipated overtime and are unlikely to cause permanent modifications to the phenotype.

**Figure 4:**
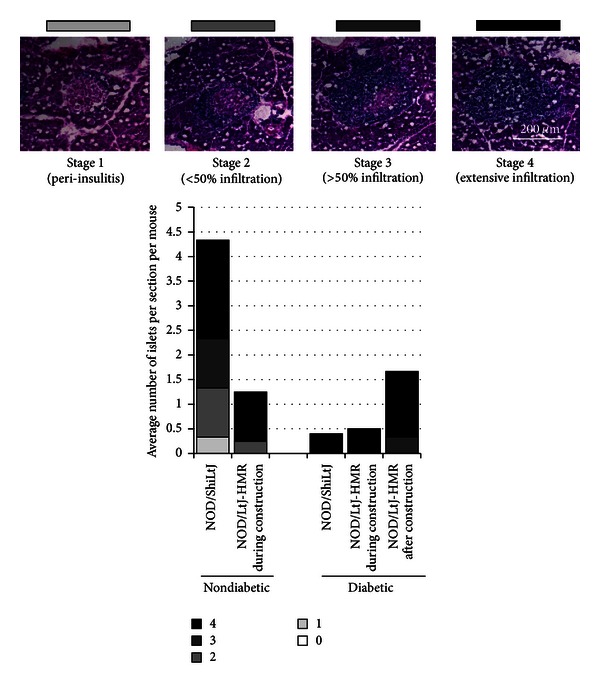
Islet destruction and lymphocytic infiltration in nondiabetic NOD mice subject to construction stress. The number of islets per pancreatic section was scored as exemplified by the histology sections presented (scale 200 *μ*m). At least three mice per group were included except for the diabetic NOD/LtJ-HMR mice during construction, which included only two mice. Note that there are no nondiabetic mice from the NOD/LtJ-HMR mice born after construction.

As mentioned previously, of the seven NOD female offspring born from mothers subject to construction stress during gestation, only two mice progressed to overt diabetes. Therefore, the environmental stress imposed by the construction during embryonic development appears to delay, but not necessarily impede, the autoimmune reaction towards pancreatic islet antigens. As such, we evaluated the subclinical progression of autoimmunity by quantifying the degree of insulitis and islet cell destruction. As expected, all diabetic NOD mice presented with few pancreatic islets and heavy lymphocyte infiltrates, suggesting an active autoimmune process (Figure 4). In contrast, the 32-week-old nondiabetic NOD/ShiLtJ mice presented with a greater number of pancreatic islets and with fewer lymphocytic infiltrates than the diabetic NOD mice (Figure 4). Surprisingly, few pancreatic islets were found in the nondiabetic NOD/LtJ-HMR mice which were subject to construction stress during embryonic development (Figure 4). Moreover, the few remaining pancreatic islets were heavily infiltrated with lymphocytes (Figure 4). These results suggest that the stress imposed by the construction did not prevent the onset of the autoimmune response and that the mice were slowly progressing towards overt diabetes.

**Figure 5:**
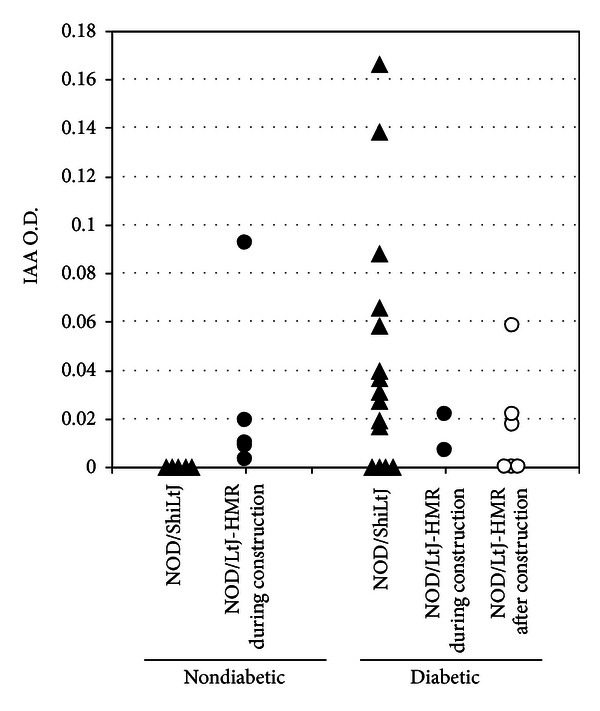
Serum IAA levels as an indication of an ongoing autoimmune response towards pancreatic islets. The serum IAA levels are shown for every mouse group and segregated according to the health status (i.e., diabetic versus nondiabetic). Note that there are no nondiabetic mice from the NOD/LtJ-HMR mice born after construction. Each dot represents one mouse.

To further define whether an active autoimmune response was ongoing in the five nondiabetic NOD/LtJ-HMR mice which were subject to construction stress during embryonic development, we determined the serum insulin autoantibody (IAA) levels. Serum IAA levels correlate with autoimmune diabetes onset in both humans and NOD mice and the serum IAA levels eventually decline with disease progression [18, 24, 25]. Expectedly, the 32-week-old nondiabetic NOD/ShiLtJ mice did not show detectable levels of serum IAA levels, while the diabetic NOD mice exhibited a variable range of serum IAA (Figure 5). In agreement with the histological observations suggestive of an active autoimmune response in the nondiabetic NOD/LtJ-HMR mice, IAA were present in the serum of these mice (Figure 5). Taken together, these results suggest that the nondiabetic NOD/LtJ-HMR mice were likely progressing towards overt diabetes. The stress imposed by the nearby construction during the embryonic development, therefore, does not preclude the onset of an autoimmune response towards pancreatic islets, although it significantly delays the progression to overt diabetes.

Limits of this current study include the low number of mice analyzed in our cohorts and the difficulty in reproducing similar events. However, similar variations in diabetes incidence have previously been documented by the Jackson Laboratory in much larger NOD animal cohorts. Indeed, the Jackson Laboratory has previously reported a modest effect of nearby construction on the incidence of diabetes in their NOD/ShiLtJ mouse colony (http://type1diabetes.jax.org/images/fine-mapping/1976%20cumulative%20inc.jpg). In contrast, we report a striking and significant delay in disease onset as well as in the cumulative incidence of disease. The main difference between these two studies lies in the stratification of data. Whereas we stratified the mice according to whether the mothers were subject to construction stress during gestation or not, the data reported by the Jackson Laboratory presents a cumulative incidence study of a larger cohort over an entire year and they did not stratify their data relative to construction events. It is thus likely that the reason we observe a more striking effect of nearby construction on both the onset and incidence of diabetes is due to a stricter stratification of affected mice. To that effect, in our hands, the nearby construction only affected mice that were born from mothers subject to construction stress during gestation and not from mice born from the same parents at a later date. The effect is thus not permanent and unlikely to cause permanent changes in a mouse colony. Together with the results of the larger NOD mouse cohort study from the Jackson Laboratory, the current study performed on a limited number of NOD mice supports the view that nearby construction can significantly affect the incidence of diabetes.

Another factor which could have influenced the incidence of diabetes in our study includes variations in the microbiome of the mouse colony over time. The animal house barrier status is known to influence the incidence of diabetes in NOD mice [1]. More recently, the microbiome was shown to influence diabetes incidence by causing variations in testosterone hormone levels and other metabolomic changes [11]. We have not directly characterized the microbiome or the hormone levels of our NOD mouse colony before and after construction. However, the routine sentinel health report status did not vary overtime, nor did the feeding, water, bedding, or procedures for the animal handling (as described in the Methods section). In addition, the fertility of NOD mice was not apparently affected by the construction stress (Table 1), suggesting that the potential changes in hormone levels were insufficient to affect the fertility. Admittedly, these represent very crude and indirect measures of the microbiome and hormone levels, and thus we cannot fully exclude the possibility that the variations in diabetes incidence in our study were due to modifications in these parameters. Of interest, changes in the microbiome have been reported to significantly alter testosterone levels without affecting NOD mouse fertility [11].

In addition to the microbiome and hormone levels, elevated serum IAA levels from the mothers have also been shown to significantly impact diabetes incidence [26, 27]. At postpartum, the NOD mothers were 10 and 15 weeks of age for the first litters subject to construction stress (Table 1). The serum IAA levels typically peak at 8 weeks of age and slowly wane off overtime [18], suggesting that the mothers were likely to present detectable serum IAA levels. Notably, first litters of NOD mothers in our animal house facility typically present with a normal incidence of diabetes (not shown). Still, the fact that we did not monitor the serum IAA levels from the mothers presents a limitation of our study. 

In summary, many parameters can influence diabetes incidence in NOD mice and there are possible alternative explanations as to why the mouse cohorts subject to construction stress during gestation showed a reduced incidence of diabetes. Nevertheless, the fact that litters born from the same mother, where one litter was subject to construction stress and the other was not, exhibited a significant difference in diabetes incidence suggests that construction stress during gestation modulates biological responses. Our results thus add to the observations from the Jackson Laboratory documenting a potential influence of nearby construction on the incidence of diabetes in NOD mice.

## 4. Conclusions

Altogether, these results emphasize the importance of surrounding factors which should be taken into consideration when performing long-term *in vivo* studies and are in agreement with the hypothesis that both environmental and genetic cues contribute to autoimmune diabetes development. Our data lend further support to the view that environmental stress caused by nearby construction severely impacts biological processes. Of interest, NOD mice suffer from significant hearing loss [28, 29] suggesting that vibrations, rather than noise, emitted as a consequence of the nearby construction provoke physiological changes in rodents. We would thus caution investigators that, prior to undertaking long-term *in vivo* studies, alterations in the surrounding environment must be carefully considered and appropriately controlled. 

## Figures and Tables

**Table 1:** Description of the mothers and litters from the NOD/LtJ-HMR mouse colony used in this study.

Breeding pair (#)	Date of birth of the mother	Became diabetic	Number of litters produced	In gestation during construction	Age of mothers at postpartum, first litter	Litters included in the study
142	June 16, 2010	Unknown	2 litters	Yes	15 weeks	1st litter born October 1, 2010
143	June 16, 2010	Yes	6 litters	No	8 weeks	N/A, not included
144	July 25, 2010	Yes	5 litters	Yes	10 weeks	1st litter born October 5, 2010 After construction born Dec 9 2010

## References

[1] Anderson MS, Bluestone JA (2005). The NOD mouse: a model of immune dysregulation. *Annual Review of Immunology*.

[2] Lehuen A, Diana J, Zaccone P, Cooke A (2010). Immune cell crosstalk in type 1 diabetes. *Nature Reviews Immunology*.

[3] Kornete M, Piccirillo CA (2011). Critical co-stimulatory pathways in the stability of Foxp3^+^ Treg cell homeostasis in Type I diabetes. *Autoimmunity Reviews*.

[4] Barrett JC, Clayton DG, Concannon P (2009). Genome-wide association study and meta-analysis find that over 40 loci affect risk of type 1 diabetes. *Nature Genetics*.

[5] Wicker LS, Todd JA, Peterson LB (1995). Genetic control of autoimmune diabetes in the NOD mouse. *Annual Review of Immunology*.

[6] Wicker LS, Clark J, Fraser HI (2005). Type 1 diabetes genes and pathways shared by humans and NOD mice. *Journal of Autoimmunity*.

[7] Knip M, Veijola R, Virtanen SM, Hyöty H, Vaarala O, Åkerblom HK (2005). Environmental triggers and determinants of type 1 diabetes. *Diabetes*.

[8] El-Osta A, Brasacchio D, Yao D (2008). Transient high glucose causes persistent epigenetic changes and altered gene expression during subsequent normoglycemia. *The Journal of Experimental Medicine*.

[9] Singh B, Rabinovitch A (1993). Influence of microbial agents on the development and prevention of autoimmune diabetes. *Autoimmunity*.

[10] Wen L, Ley RE, Volchkov PY (2008). Innate immunity and intestinal microbiota in the development of Type 1 diabetes. *Nature*.

[11] Markle JG, Frank DN, Mortin-Toth S (2013). Sex differences in the gut microbiome drive hormone-dependent regulation of autoimmunity. *Science*.

[12] Williams AJK, Krug J, Lampeter EF (1990). Raised temperature reduces the incidence of diabetes in the NOD mouse. *Diabetologia*.

[13] Funda DP, Kaas A, Bock T, Tlaskalova-Hogenova H, Buschard K (1999). Gluten-free diet prevents diabetes in NOD mice. *Diabetes/Metabolism Research and Reviews*.

[14] Hoorfar J, Buschard K, Dagnaes-Hansen F (1993). Prophylactic nutritional modification of the incidence of diabetes in autoimmune non-obese diabetic (NOD) mice. *British Journal of Nutrition*.

[15] Furuse M, Kimura C, Takahashi H, Okumura JI (1991). Prevention of the incidence of diabetes by dietary sorbose in nonobese diabetic mice. *Journal of Nutrition*.

[16] Dugas V, Beauchamp C, Chabot-Roy G, Hillhouse EE, Lesage S (2010). Implication of the CD47 pathway in autoimmune diabetes. *Journal of Autoimmunity*.

[17] Pozzilli P, Signore A, Williams AJK, Beales PE (1993). NOD mouse colonies around the world—recent facts and figures. *Immunology Today*.

[18] Yu L, Robles DT, Abiru N (2000). Early expression of antiinsulin autoantibodies of humans and the NOD mouse: evidence for early determination of subsequent diabetes. *Proceedings of the National Academy of Sciences of the United States of America*.

[19] Bonifacio E, Atkinson M, Eisenbarth G (2001). International Workshop on Lessons From Animal Models for Human Type 1 Diabetes: identification of insulin but not glutamic acid decarboxylase or IA-2 as specific autoantigens of humoral autoimmunity in nonobese diabetic mice. *Diabetes*.

[20] Babaya N, Liping Y, Dongmei M (2009). Comparison of insulin autoantibody: polyethylene glycol and micro-IAA 1-day and 7-day assays. *Diabetes/Metabolism Research and Reviews*.

[21] Baxter AG, Hamilton F, Mandel TE, Augustine C, Cooke A, Morahan G (1993). Genetic basis for diabetes resistance in NOD/Wehi mice. *European Journal of Immunogenetics*.

[22] Baxter AG, Koulmanda M, Mandel TE (1991). High and low diabetes incidence Nonobese Diabetic (NOD) mice: origins and characterisation. *Autoimmunity*.

[23] Baxter AG, Adams MA, Mandel TE (1989). Comparison of high- and low-diabetes-incidence NOD mouse strains. *Diabetes*.

[24] Steck AK, Johnson K, Barriga KJ (2011). Age of islet autoantibody appearance and mean levels of insulin, but not GAD or IA-2 autoantibodies, predict age of diagnosis of type 1 diabetes: diabetes autoimmunity study in the young. *Diabetes Care*.

[25] Bonifacio E, Ziegler AG (2010). Advances in the prediction and natural history of type 1 diabetes. *Endocrinology and Metabolism Clinics of North America*.

[26] Greeley SAW, Katsumata M, Yu L (2002). Elimination of maternally transmitted autoantibodies prevents diabetes in nonobese diabetic mice. *Nature Medicine*.

[27] Melanitou E, Devendra D, Liu E, Miao D, Eisenbarth GS (2004). Early and quantal (by litter) expression of insulin autoantibodies in the nonobese diabetic mice predict early diabetes onset. *Journal of Immunology*.

[28] Atkinson M, Gendreau P, Ellis T, Petitto J (1997). NOD mice as a model for inherited deafness. *Diabetologia*.

[29] Lonyai A, Kodama S, Burger D, Faustman DL (2008). Fetal Hox11 expression patterns predict defective target organs: a novel link between developmental biology and autoimmunity. *Immunology and Cell Biology*.

